# Medical, Surgical, and Hygienic Exhibition

**Published:** 1898-06-18

**Authors:** 


### MEDICAL, SURGICAL, AND HYGIENIC EXHIBITION.

(*Continued from page* 192).

The Aylesbury Dairy Company (Limited) occupied a centre stall of unique appearance. The most interesting compositions of milk were on view.

Mellin's Food Company (Limited) and Mellin's Emulsion Company (Limited).—The stall was one of the centres of rendezvous, these companies exhibiting their various preparations, which possess so world-wide a reputation.

Down Bros. exhibited a large selection of surgical instruments, giving evidence of their ability to meet the requirements of modern surgery in its various branches. Space does not admit of a full description of this exhaustive exhibit. However, we would draw attention to some striking advances in instruments for bone screwing, made for Mr. Arbuthnot Lane, a mouth gag by Dr. Cock, syringes for hypodermic injections, &c., made entirely of metal, and some very completely-arranged surgical and midwifery bags.

L. Rose and Co.—The stand of these manufacturers of "Rose's Lime Juice Cordial," which is a well-known beverage for summer-time, attracted much attention.

Horlick and Co. displayed to advantage their Horlick's Malted Milk, which has now acquired a reputation in all parts of the world.

Bovril (Limited).—This nicely arranged stall, under the personal superintendence of Dr. Moxon, was constantly surrounded by medical men and nurses, who evidenced much interest in "Bovril."

Parke, Davis and Co.—This firm made a very attractive display of pharmaceutical preparations, amongst which special attention must be drawn to their egg emulsion, cod liver oil, taka-diastase, cascara sagrada, and anti-diphtheritic serum, as well as their hypodermic syringe in a handsome aluminium case, fitted with needles and immediately soluble tablets, each containing the appropriate dose of different medicaments.

W. H. Bailey and Son were showing a large variety of the newest instruments and appliances for sick nursing, including well-made light trusses and elastic stockings of the finest texture. Their aseptic ward table of plate glass and enamelled iron appeared to us to be very reasonable in price.

Henri Nestlé.—On this stand, besides the ordinary Nestlé's milk and food, was shown the "Viking" brand unsweetened condensed milk, manufactured in Norway, and taken over by Mr. Henri Nestlé since the beginning of the year.

Oppenheimer, Son, and Co. (Limited). — The special features of this handsome stall were the show of palatinoids and bi-palatinoids of animal substances, powdered drugs, and essential principles; hypodermic cases, cream of malt, and concentrated liquors. This firm recently received the gold medal at the International Medical Congress.

Jeyes' Sanitary Compounds Company (Limited) exhibited their products in a lavish manner. These included "Creolin" in various forms, and a new preparation known as "Branalcane," to be used in throat affections. Their automatic toilet paper distributing box is a decided advantage over the ordinary paper in rolls.

The Fairchild Digestive Products (agents, Burroughs, Wellcome, and Co.).—The three most recent products specially displayed were "Peptogenic Milk Powder" for modifying cow's milk to the standard of human milk; "Panopepton," the peptonised nutrient of beef and bread preserved in brown sherry; and "Pepsensia," an agreeable digestive fluid. This stand attracted considerable attention.

Alfred Cox, of 108, New Bond Street, had a good selection of aseptic instruments and appliances, also nursing requirements for hospitals and sick-rooms.

The Hovis Bread Flour Company (Limited).—Besides the "Hovis" bread, this company showed all kinds of pastry and fancy cakes recently introduced, and made from the same flour.

Vejos (Limited).—This company's powerful nutrient (*Lancet*, April 16th, 1898) was exhibited prominently at their stall. All day long doctors and nurses were to be seen tasting "Vejos."

Fleming's Oil and Chemical Company (Limited).—The latest speciality to be seen as manufactured by this firm was the "Ammoniated Camphylene Cream" for bath and toilet use, and also for domestic purposes as a disinfectant.

Nutroa (Limited) exhibited their Nutroa food, claimed to be a starch-free food, with the fat and albuminoids adjusted so that, when prepared, it is identical with human milk.

Madame Mona and Co.'s stall was interesting in the display of sanitary sheets and towels. A practical demonstration of the efficacy of their waterproof sheeting was given at the stall.

The Valentine Extract Company (Limited).—This company's novelty—the "Valtine" Meat Globules—drew considerable attention, owing to the fact that beef tea can be made at a moment's notice. Sample cups were freely distributed.

The Scientific Press (Limited), in addition to showing works on medicine and surgery, made a considerable display of books dealing with hospitals and nursing.

Liebig's Extract of Meat Company (Limited).—This company's usual artistic stand was to be seen, showing their well-known "J. V. Liebig Extract" and their new invalid food "Peptarnis," which attracted a large share of patronage from the number of medical men in attendance.

Cooper and Co., of South Kensington, prominently displayed an assortment of new chemical products. These chemists have recently introduced the new patented oxycarbonated mineral waters.

E. Beanes and Co. made a special show of their food for infants, invalids, and elderly people. It is claimed for this food that it contains a large percentage of diastase from malted barley.

Hall's Wine stand was one of the most tastefully arranged. The wine itself needs no comment, as it is now so well known to the medical profession. The proprietors presented to every medical man attending the exhibition an engraving, after Cousins, of Sir Astley Paston Cooper, Bart., F.R.S. This engraving in itself was an appreciable memento of the exhibition.

M. Hoff, of Hamburg (Leopold Hoff, proprietor).—At this stall was shown a new preparation of Hoff's Malt Extract, with manganese and iron. The extensive manner in which the malt extract itself is prescribed by the medical profession should augur well for its future.

S. Maw, Son, and Thompson.—Their display of surgical instruments and antiseptic dressings was one of the features of the exhibition. Their most recent improvement is a brass and glass instrument case for use in the operation theatre, absolutely air-tight and dust-proof.

